# Thermocapillary central lamella recess during droplet impacts onto a heated wall

**DOI:** 10.1038/s41598-024-51382-3

**Published:** 2024-01-11

**Authors:** Patrick Palmetshofer, Anne K. Geppert, Jonas Steigerwald, Tim Arcos Marz, Bernhard Weigand

**Affiliations:** Institute of Aerospace Thermodynamics, University of Stuttgart, 70569 Stuttgart, Germany

**Keywords:** Fluid dynamics, Thermodynamics

## Abstract

We experimentally observe a new phenomenon, the formation of a toroidal region of lower film thickness in the center of the lamella formed during high Weber number water droplet impacts onto smooth heated walls. This region forms around the air bubble, which is entrapped during the initial impact phase at the impact center. Our study encompasses a variation of the droplet size, impact velocity, surface wettability and temperature. We show how this phenomenon can be explained considering a two-step process involving thermocapillary convection in two separate regions: The temperature gradient along the surface of the entrapped air bubble caused by heat conduction induces flow that pumps warmer liquid to the lamella-ambient interface due to the Marangoni effect. The non-uniform temperature distribution along it then causes fluid acceleration in the radial direction, depleting the fluid volume around the bubble in a self-amplifying manner. We use direct numerical simulations of a stagnant liquid film with an enclosed bubble at the wall to confirm this theory.

## Introduction

Droplet impact dynamics onto heated surfaces have been studied extensively in the past as they are crucial in understanding the underlying physics of processes such as spray cooling of high-power electronics, steel quenching and fuel droplet impacts onto combustion chamber walls in rocket engines^1–5^. The outcome of a droplet impact is affected by factors such as the surface wettability, the fluid properties and the geometric parameters of the impacting droplet. However, the influence of the surface temperature $T_w$ is of special interest for these applications^6–8^ due to its relevance in the heat transfer processes. Four different heat transfer regimes are usually defined: film evaporation, nucleate boiling, transition boiling and film boiling^7,9^. Previous research focused mainly on the nucleate and film^10,11^ boiling regimes, especially near the Leidenfrost point^12^. In contrast, the evaporation regime, which is defined by a surface temperature below the saturation temperature $T_{sat}$ of the liquid, was mainly studied for sessile droplets^13^.

**Figure 1:**
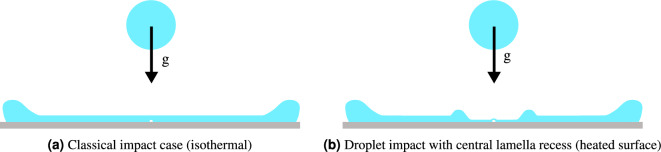
Illustration of the difference between an impact case with and without the central lamella recess.

Hydrodynamically, the droplet impact process can be split into four phases: the kinematic, spreading, relaxation and wetting equilibrium phase^14^. During the kinematic phase an air bubble is entrapped at the point of impact of the droplet^15–18^. When the droplet approaches the wall, an air cushion is formed between the droplet and the solid surface while the droplet assumes a shape of a truncated sphere. At some point, the viscosity of the air suppresses the displacement of this air layer and the liquid first contacts the surface in an annular ring shape. The now enclosed air film rapidly retracts into a spherical-cap shaped bubble as it minimizes its surface energy^19–21^. Figure 1a shows a schematic of a droplet impact at the end of the spreading phase with this enclosed air bubble and a uniformly thick liquid lamella. Observing the entrapped air bubble with total internal reflection (TIR) imaging, the Laser Pattern Shift Method^22^ can be used to measure the liquid layer thickness at the center of the spreading lamella^23^ from the double shadow cast by the bubble. The rupture of this air bubble can significantly affect the impact dynamics during spreading or relaxation phase: It can cause the ejection of a violent jet^24^ or the formation of a dry spot around the impact center^24,25^, which strongly influences the receding and rebound behavior.

Our investigation revealed a new phenomenon which only occurs in droplet impacts onto heated surfaces: this bubble can cause a *central lamella recess* as shown in Fig. 1b, which leads to film thicknesses smaller than the height of the bubble. We suggest that thermocapillary (Marangoni) effects at this enclosed air bubble can cause a non-uniformity in the surface temperature of the lamella formed during droplet impact, which then causes outward Marangoni flow. If the bubble bursts during the expansion of the recess zone, the center of the lamella dewets, quickly engulfing the entire recess zone. Similar behavior was previously observed for stagnant films^26,27^ and bubbles on a heated wall^28–33^. The additional flow induced by this phenomenon could increase the heat transfer in the central region or cause dewetting and could thus be relevant to future spray cooling models and coating processes.

In this study, we dropped water droplets onto a uniformly heated surface at various temperatures below the saturation temperature $T_{w}<T_{sat}$ of either close to ambient temperature (no heating) and constant values of ${323}\,\text{K}$, ${343}\,\text{K}$, ${363}\,\text{K}$, or ${393}\,\text{K}$. We conducted our experiments using a four-perspective (top, lateral, bottom, spatial) high-speed imaging setup employing diffuse back-light and total internal reflection imaging^23^. The smooth sapphire glass samples used as the impact target were either left hydrophilic (contact angle $\theta < 40^{\circ }$) or were hydrophobized ($\theta \approx 120^{\circ }$) to account for the effect of the surface wettability. To characterize the hydrodynamic impact behavior, the Weber number $\text {We}=\rho d u^2/\sigma$ and Reynolds number $\text {Re}=d u/\nu$ are commonly used^14^. Here, *d* and *u* are the initial droplet diameter and impact velocity, respectively, $\rho$ is the density, $\sigma$ is the surface tension and $\nu$ is the kinematic viscosity of the liquid. To investigate the effect of Re and We, we released water droplets of two different diameters (${2.11}\,\text{mm}$ and ${2.95}\,\text{mm}$) and varied the falling height between ${0.15}\,\text{m}$ and ${1.3}\,\text{m}$, corresponding to Weber numbers between 80 and 800 and Reynolds numbers between 4,000 and 14,500. Fluid properties at room temperature are used to estimate $\text {We}\,\text {and}\,\text {Re}$. The ambient temperature was measured to vary between ${296}\,\text{K}$ and ${298}\,\text{K}$.

In the following, the *central lamella recess* is described in detail and the effect of surface temperature $T_{w}$ and impact Weber number $\text {We}$ on its appearance is classified. In addition, an overview of the recess formation process is given. Afterwards, we present a short summary of the suggested two-step thermocapillary convection mechanism causing the recess. In order to support our explanation, the evolution of the central lamella thickness was measured before and during the recess formation. In a second step, the derived thickness is utilized to initialize numerical simulations of a stagnant thin liquid film heated from below containing an enclosed air bubble.

## Results

### Classification of the central lamella recess

**Figure 2:**
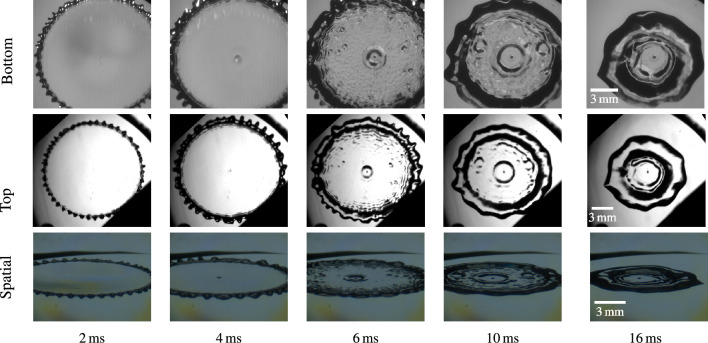
*Central lamella recess* formation after a water droplet ($d = {2.95}\,\text{mm}$) impact from $h={0.7}\,\text{m}$ ($\text {We}=480$, $\text {Re}=11,800$) onto a surface heated to ${363}\,\text{K}$. The timestamp of the spatial view can vary by $\pm {0.2}\,\text{ms}$, due to the lower frame rate.

The temporal development of the newly observed *central lamella recess* phenomenon is shown in Fig. 2 for a water droplet impact onto a surface heated to ${363}\,\text{K}$ with a Weber number of $\text {We}=480$ and a Reynolds number of $\text {Re}=11,800$. At ${2}\,\text{ms}$ after impact, which corresponds to a time during the lamella spreading phase of the droplet impact process^14^, the impact bubble is visible in all three views, including a double shadow in the bottom view (see section on lamella thickness for an explanation of the double shadow^23^). At this point, there is no significant phenomenological difference to cases in which the surface remains near ambient temperature. However, at the beginning of the relaxation phase^14^ at ${4}\,\text{ms}$, a disturbance of the film around the central impact bubble is visible, indicating a curved dip in the lamella height above the bubble. At this time, the two shadows of the impact bubble seen in the bottom view are still distinguishable, but already overlap. After this time, they rapidly collapse into a single shadow with a curved region around it, while the previously formed dip develops into a larger, circular recess region. At ${6}\,\text{ms}$, the curved region around the impact bubble appears as a larger dark spot in all three views shown in Fig. 2. In this frame, the recess region has already expanded slightly and further expands until ${16}\,\,\text{ms}$ after impact. During its expansion, the recess region around the impact bubble remains calm, seemingly unaffected by the oscillations outside of this region and even resists incoming capillary waves. This is apparent in the frames taken ${16}\,\text{ms}$ after the initial impact. Interestingly, even between ${10}\,\text{ms}$ and ${16}\,\text{ms}$ after impact, the recess region further expands with its expansion speed reducing gradually, while the outer rim of the impacting droplet already retracts. Finally, after ${16}\,\text{ms}$, the retraction slowly propagates into the recess region until it disappears during the droplet relaxation phase after approximately ${22}\,\text{ms}$. Apart from the central recess, Fig. 2 also shows similarly looking dips in other locations in the lamella at ${6}\,\text{ms}$, although only two such areas remain after ${10}\,\text{ms}$. This suggests that a film instability mechanism is responsible for the recess phenomenon which is amplified or catalyzed by the central impact bubble.

**Figure 3:**
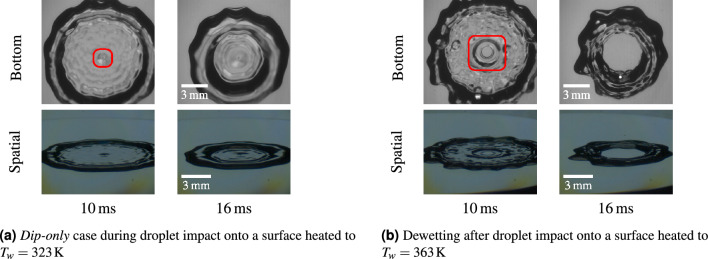
Other outcomes of droplet impacts from a falling height of $h={0.7}\,\text{m}$ with a droplet diameter of $d = {2.95}\,\text{mm}$ ($\text {We}=480, \text { Re}=11,800$).

In some observed cases, only the initial disturbance of the film seen at ${4}\,\text{ms}$ in Fig. 2 is observed, but not the rapid expansion of the recess region of low film height. An example of such a case is shown in Fig. 3a, which differs from the previously discussed case only in the surface temperature, which is set to ${323}\,\text{K}$ instead of ${363}\,\text{K}$. Generally, with a lower surface temperature, the onset of the phenomenon happens later and its strength is reduced. In the following, we denote such cases where the dimple is formed, but no rapid lamella recess is observed as *dip-only* cases. These are characterized by the two bubble shadows in the total internal reflection view remaining distinguishable throughout the whole spreading process.

In many cases, such as the one shown in Fig. 2, the thinned lamella remains stable and only disappears after the incoming receding rim washes over it. Figure 3b shows another possibility for the later stage outcome of the impact. In this case, the bubble bursts during the expansion of the recess zone and the center of the lamella dewets, quickly engulfing the entire recess region. Thus, an annular ring is formed which retracts slower than a stable lamella-rim combination. However, we could not identify a consistent predictor for the occurrence and time of the dewetting or whether the thinned lamella remains stable for lower surface temperatures than the saturation temperature. Note that this type of dewetting in the center must clearly be distinguished from central dewetting induced by incoming capillary waves^24^. However, a combination of both phenomena was occasionally observed in some cases. From the observations, we assume that the central bubble, when a significant recess region has formed, presents an additional elevation above the recess film height (see schematic in Fig. 5). In this case, the bubble is separated from the air by a thin film only, similar to a soap bubble in air. Dewetting of the *central lamella recess* region also always occurs in the cases where the surface temperature is set to ${393}\,\text{K}$. However, nucleate boiling and the formation of additional dewetted regions prevents the formation of a toroidal ring (See video 6 in supplementary materials).

**Figure 4:**
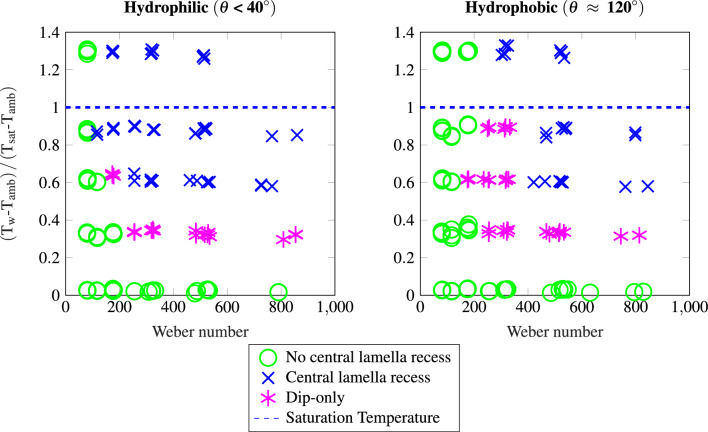
Classification of impact outcomes (lamella recess, no lamella recess and *dip-only* cases) of water droplet impacts onto hydrophilic (left) and hydrophobic (right) surfaces at various impact Weber number and dimensionless surface temperature.

Figure 4 shows a regime map of the observed cases, separating droplet impacts where no recess formation is observed from those where only the initial disturbance can be seen (*dip-only*) from the cases where the recess is formed. Whether the droplet forms a *central lamella recess* seems to be determined by both the impact Weber number as well as the temperature to which the surface is heated. Note that the lowest row of points represents no intentional surface heating, but the high-power LEDs increase the surface temperature by $\Delta T \approx {2}\,\text{K}$ over ambient temperature. For these cases, the observation of no recess formation is consistent with existing works at very similar conditions in a comparable experimental setup^22^. Also, even for heated surfaces, the *central lamella recess* does not occur at low impact energies up to a certain threshold Weber number. For the hydrophobic cases, the threshold Weber number for which first signs of the recess (*dip-only*) appear is above $\text {We} > 200$ at ${323}\,\text{K}$ surface temperature ($\left( T_w-T_{amb}\right) /\left( T_{sat}-T_{amb}\right) \approx 0.33$), and $\text {We} > 100$ at ${363}\,\text{K}$ ($\left( T_w-T_{amb}\right) /\left( T_{sat}-T_{amb}\right) \approx 0.87$). For the hydrophobized surfaces with a contact angle of $\theta \approx 120^{\circ }$, the thresholds are shifted towards higher Weber numbers, albeit the temperature dependence appears comparable. Comparatively observing the video data at impact conditions where a phenomenological difference between the hydrophilic and hydrophobic surfaces was observed, reveals that the initial behavior in the center of the droplet is similar. However, in case of impacts onto hydrophobic surfaces, the maximum spreading diameter and thus the relaxation phase of the droplet impact is reached earlier^22^. This causes an earlier arrival of capillary waves from the droplet rim which can interrupt the formation of the recess region, especially if the formation is still in an early stage. This suggests that whether a lamella recess forms could be a function of the residence time and thickness of the film. The dependence of the occurrence of the *central lamella recess* on the surface contact angle could result from both the residence time until the first capillary waves reach the impact center in the relaxation phase and the shape of the bubble: a higher liquid-solid contact angle could cause a flatter central impact bubble.

### Central lamella recess induced by two-step thermocapillary convection

**Figure 5:**
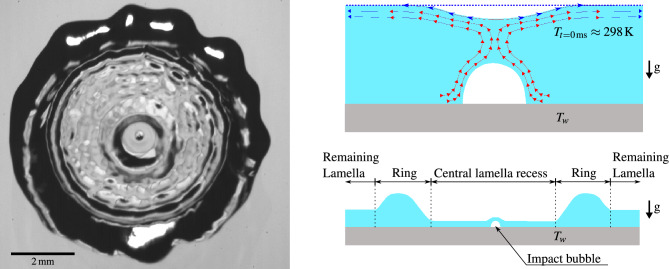
Image at $t={5}\,\text{ms}$ of the observed surface recess for a droplet impact case with $\text {We}=320$, $\text {Re}=7700$ onto a surface at ${363}\,\text{K}$ and schematic of the two-phase recess formation, with thermocapillary induced flow paths.

As no *central lamella recess* was observed without heating, both in our experiments and a similar study^22^, heat transfer through the liquid lamella must be directly or indirectly responsible for the phenomenon. Notably, the recess region grows to an order of magnitude larger than the enclosed bubble, but we experimentally observe that the bubble does not expand significantly at surface temperatures below $T_s$. We found that the recess formation is similar to the formation of a dry spot when the center of a thin liquid film is selectively heated to a higher temperature than an outer region^26^ or when a pendant isopropanol droplet is placed above a thin water film^27^. Both of these effects were reported to result from the Marangoni effect due to temperature (thermocapillary flow) or concentration gradients (solutocapillary flow), respectively. This suggests that an increased temperature of the lamella-ambient interface in the center of the droplet may cause the *central lamella recess*. Expanding on this idea we hypothesize that the lamella recess is caused by thermocapillary convection in two steps at separated interfaces: The central bubble and the lamella-ambient interface (see Fig. 5). During the initial phase after impact, heat is conducted into the fluid in the near-wall region, but no significant temperature change happens at the top of the lamella. However, the enclosed central bubble now presents a free surface with a significant tangential temperature gradient. Thus, heated fluid from the near-bubble/near-wall region is transported along the interface of the bubble. As this process is symmetric, the flow of heated water has to separate from the bubble and causes a local temperature increase above the sessile bubble. Very similarly, thermocapillary flow emanating from a vapor bubble on a heated wall has been observed in several studies^28–31^. Therefore, hot fluid is transported from the near-wall region towards the top of the lamella. This, in turn, causes a local temperature increase on the film-ambient interface above the bubble, while no such increase happens far away from the bubble, causing a radial temperature gradient. This marks the beginning of the second step of the process: Thermocapillary flow is now induced radially along the lamella-ambient interface. Due to the low film thickness, viscosity suppresses the recirculatory flow at the bottom of the flow and the lamella height decreases above the bubble. Due to the persisting vertical temperature gradient at this timescale, when the film reduces in height in the near-bubble region, the radial temperature gradient is increased, further accelerating the film depletion process. This effect can thus cause the film height next to the bubble to reduce even below the height of the bubble, where heat conduction has already heated up the fluid. Figure 5 depicts an image of the *central lamella recess* at $t={5}\,\text{ms}$ after impact for a droplet impact case at $\text {We}=320$, $\text {Re}=7700$ and a surface temperature of $T_w={363}\,\text{K}$. Additionally, a schematic of the *central lamella recess* and the formed inner ring of higher film thickness is shown. The schematic also shows that the bubble top remains above the newly reduced lamella height in the recess region, which is why a larger region is visible around the bubble once the lamella recess occurs. As the thermocapillary pumping of liquid from the bottom layer to the top is self-damping, it can only significantly affect the lamella top if the latter quickly reaches a height of similar magnitude as the bubble size. As reported in literature^34,35^, a higher impact Weber number reduces the residual film height, suggesting the existence of a threshold Weber number.

### Lamella thickness in the recess region

**Figure 6:**
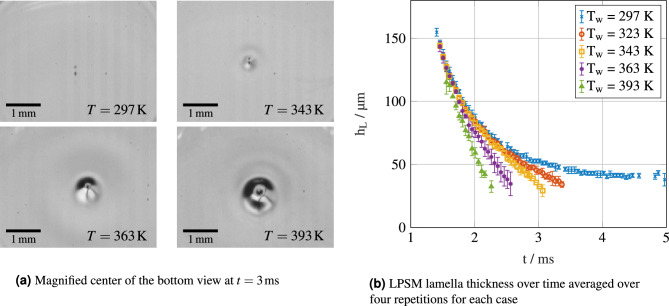
Magnified view of the bubble shadow from the bottom view at $t={3}\,\text{ms}$ (**a**) and LPSM^23^ film thickness (**b**) for impacts from $h={0.7}\,\text{m}$ with a droplet diameter of $d = {2.11}\,\text{mm}$ ($\text {We}=320,\text {Re}=7,700$).

To determine whether the hypothesized mechanism can lead to the observed lamella recess, we check whether the bubble size is of similar magnitude as the film height before the occurrence of the phenomenon. To do this, our total internal reflection view can be used to measure the film thickness in the center of the lamella using the Laser Pattern Shift Method (LPSM)^23^. Two half-shadows of the central bubble are cast in the total internal reflection view and the centers of the two bubble shadows are tracked using an image processing algorithm. Using the expression $h_{L,c}=d_{s}\sqrt{n_F^2/n_P^2-0.5}$, the lamella thickness in the center $h_{L,c}$ can be calculated from the distance $d_{s}$ between the bubble shadows on the camera, the refractive indices of the fluid $n_F$ and the prism $n_P$^23^. Figure 6b shows the film thicknesses measured during the impact using the laser pattern shift method^23^ for a droplet dropped from ${0.7}\,\text{m}$ onto hydrophilic surfaces. While the lamella thickness converges to a value of $\approx {45}\,\upmu \text{m}$ in the non-heated case, the thickness reduces below a measurable value for the heated cases, corresponding to a coalescence of the initially separate bubble shadows forming a single dark spot in the image instead. In previous studies, the vertical length of the combined spot was used to determine the film thickness. However, in our heated cases, the combined bubble shadow quickly becomes circular, with an additional darker, larger region around it. This is shown in Fig. 6a, where four different impact cases with the same impact parameters, but different surface temperatures are displayed at ${3}\,\text{ms}$ after impact. As a higher surface temperature accelerates the occurrence of the recess phenomenon, the different temperatures also represent different stages in the formation of the recess (compare with Fig. 2). At room temperature and at ${343}\,\text{K}$, two shadows can still be clearly distinguished, but a formation of a curved “dip” region becomes apparent at higher temperatures. In the ${363}\,\text{K}$ and ${393}\,\text{K}$ cases, the double bubble shadow has already collapsed into a single, circular area, indicating a collapse of the film thickness around the bubble to a height lower than the bubble. This distinction can also be seen in Fig. 6b, where at ${3}\,\text{ms}$, no values for the measured film thickness are given for the two highest temperatures. Figure 6a also shows that in the case where the surface temperature is above the saturation temperature of water, the bubble size is increased significantly as water is evaporated into the bubble. Note that the spreading diameter at the times displayed in Fig. 6a is similar (within 10% of each other) at all temperatures, which suggests that the lamella thicknesses in regions far away from the bubble may also be less dependent on the surface temperature than the thickness measured in the center using the laser pattern shift method.

To further confirm our observations, we modified the optical setup using a higher magnification and added polystyrene particles with a diameter of ${4.98}\,\upmu \text{m}$ to the impacting droplet at a concentration of ${0.5}\,\text{g}$ per ${40}\,\text{mL}$. Adding the particles allowed to qualitatively confirm that while the radial flow velocity reduces over time in the initial impact phase^34,36^, the formation of the recess region is caused by an additional acceleration of the fluid away from the impact bubble (see video 5 in supplementary materials). Furthermore, the double particle shadows for stagnant particles (usually assumed to stick to the wall) become indistinguishable, indicating a very thin film height $< {20}\,\upmu \text{m}$ in the recess region.

Values obtained for the film thickness at different times are used to initialize idealized DNS to qualitatively confirm our theory for the recess formation and whether the film can reduce to a thickness lower than the bubble size. By measuring the bubble shadow, an estimate for the bubble diameter can furthermore be gathered, which was found to be between ${50}\,\upmu \text{m}$ and ${100}\,\upmu \text{m}$.

### Numerical simulation of a bubble in a stagnant heated film

In order to qualitatively confirm that the *central lamella recess* can happen due to the described two separate steps of thermocapillary convection at the bubble and the film interface, we employed DNS of a static film with an enclosed air bubble. We used our in-house code Free Surface 3D (FS3D)^37^ on a three-dimensional Cartesian grid with symmetry conditions through the bubble. As the measured residual film height without heating is $h_{res} \approx {45}\,\upmu \text{m}$ in the cases shown in Fig. 6b we chose a value of ${48}\,\upmu \text{m}$ for the initial film height in the simulations. The initial diameter of the bubble was set to ${64}\,\upmu \text{m}$ and the initial film temperature was set to $T_0={293.15}\,\text{K}$. Two simulations using the same setup and solver were conducted: One with a constant surface tension of $\sigma = {72.0}\,\text{N m}^{-1}$ and one using a linear, temperature-dependent model for the surface tension $\sigma (T)$. In the linear approximation, the gradient at ambient temperature $d \sigma / d T = {-0.00013}\,\text{N m}^{-1}\,\text{K}^{-1}$ obtained from surface tension tables^38^ was chosen to obtain a conservative estimate and the surface tension at a reference temperature of ${298}\,\text{K}$ was $\sigma = {72.0}\,\text{N m}^{-1}$.

**Figure 7:**
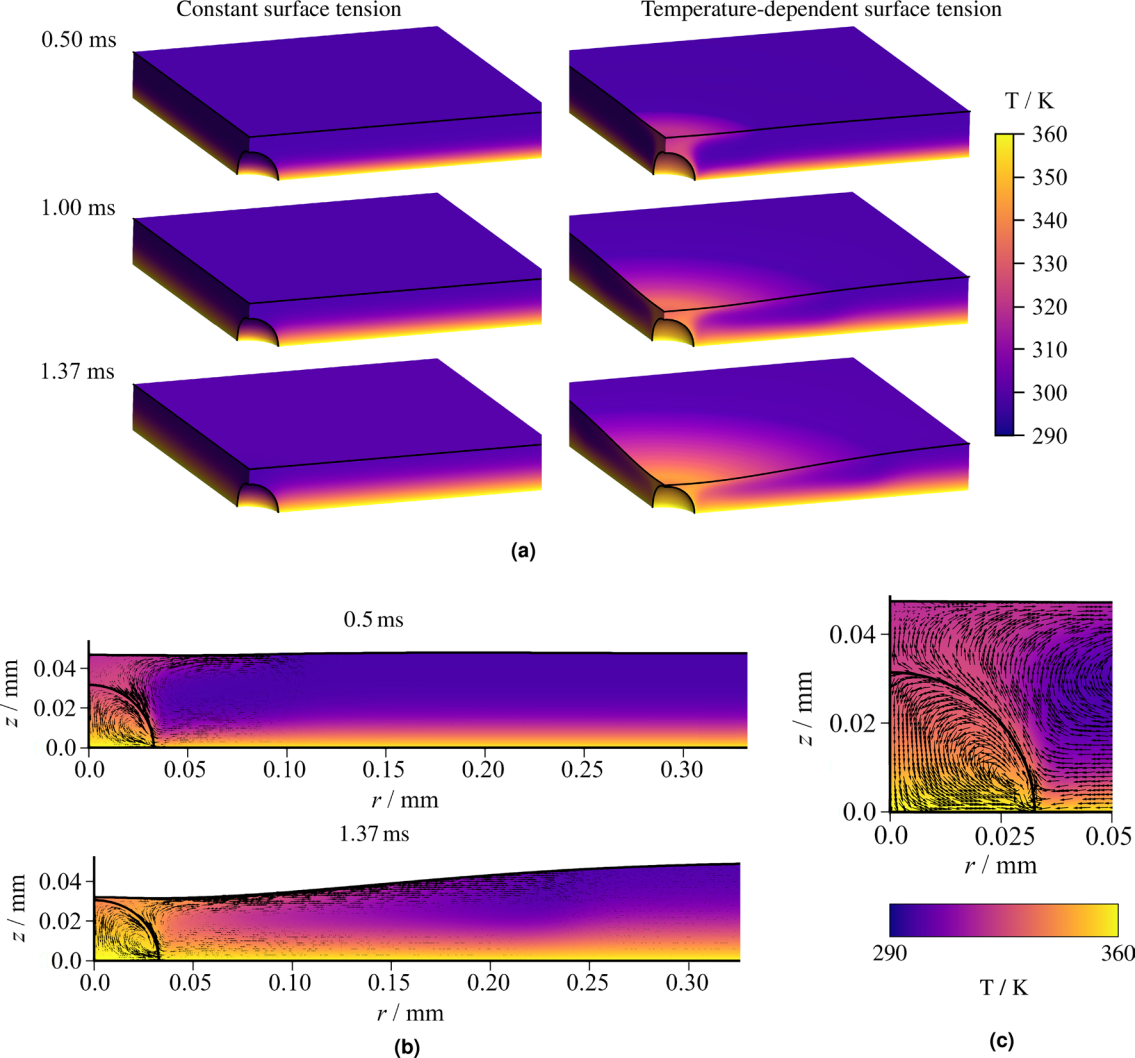
Simulation results showing the temperature distribution and gas-liquid interface (black line) for a thermocapillary-capable simulation. The initial film height and bubble diameter were set to ${48}\,\upmu \text{m}$ and ${64}\,\upmu \text{m}$, respectively, and the wall temperature was ${363}\,\text{K}$.

A comparison of the two simulations shown in Fig. 7 demonstrates that after ${0.5}\,\text{ms}$, the temperature rise caused by heat conduction, which can be seen far away from the bubble in both cases, does not yet have a significant effect on the lamella-ambient interface. However, in the vicinity of the bubble, the thermocapillary simulation shows a mushroom-cloud like shape and a region close to the bubble where the temperature gradient is increased due to the flow towards and along the bubble surface. Heat conduction through the liquid layer remains the dominant heat transfer phenomenon in the simulation with constant surface tension, while with a linear surface tension model, the thermocapillary convection along the bubble and subsequently along the lamella top dominates the flow. At ${1.0}\,\text{ms}$, the lamella-ambient interface above the bubble remains stagnant in the constant surface tension case, while the film thickness is already significantly reduced in this region in the simulation with a temperature-dependent surface tension. This reduction results from the radial thermocapillary induced flow at the top of the film. At ${1.37}\,\text{ms}$ the lowest film height in the vicinity of the bubble has dropped below the apex of the enclosed bubble. Shortly after the time displayed, the bubble bursts in the simulation with a temperature-dependent surface tension while the interface seems to remain unchanged in the constant surface tension simulation. Here, the numerical simulation may be limited by its grid resolution, as in reality, we assume that the bubble wall may attain a thickness of below ${1}\,\upmu \text{m}$ which cannot be resolved in the simulation.

## Discussion

We hypothesized that a two-step process causes the newly observed *central lamella recess* occurring in water droplet impacts onto heated walls. It arises from a transient temperature gradient at the bubble boundary, causing thermocapillary transport of hot water from the near-wall region to the lamella-ambient interface and subsequent radial thermocapillary convection at the lamella top. Our numerical simulations of a stagnant film support this hypothesis and show that the microscopic bubble entrapped in the initial impact can have significant effects on the macroscopic dynamics of the droplet impact process.

In the future, other fluids should be investigated and the effect of the presented phenomenon on the heat transfer between the fluid and the wall should be investigated. Additionally, three-dimensional numerical simulations of the complete impact process, accounting for heat transfer and thermocapillary convection could shed light onto the physical micro-mechanisms causing the phenomenon. Recent works^39–41^ have also shown that the presence of surface structures can lead to the entrapment of additional air bubbles during the droplet impact process. The effect described in this study could thus be relevant when investigating future spray cooling methods that utilize surface structures to increase the heat transfer.

## Methods

### Experimental setup

The experiments are conducted on a four-perspective (top, lateral, bottom, spatial) high-speed imaging setup employing diffuse back-light and total internal reflection imaging originally developed by Foltyn et al.^22^. The top and lateral perspective (diffuse back-light imaging) are combined onto a single Photron SA-X2 camera using a beam splitter arrangement. The bottom perspective is recorded with the second Photron SA-X2 camera in a total internal reflection setup^42^, which allows for accurate tracking of the contact line on the target surface. Both cameras record at 20,000 fps with a resolution of $1024 \times 672 \text{ px}^2$. The optical resolutions are ${17}\,\upmu \text{m}/ \text{px}$, ${18}\,\upmu \text{m}/\text{px}$ and ${28}\,\upmu \text{m}/\text{px}$ for the bottom, lateral and top view, respectively. The spatial view was recorded using a Krontech Chronos 1.4 camera, capturing 2,500 fps at a resolution of $800 \times 600 \text{ px}^2$. The image acquisition of all cameras is triggered with a laser light barrier, however, only the two Photron SA-X2 cameras are synchronized for each frame. The droplets are generated by regular drip off at the tip of a blunt, tilted needle that is fed by a medical syringe pump.

The optical glass prism originally used by Foltyn et al.^22^ was replaced by a sapphire glass prism of the same size, which is clamped between two copper plates to allow for surface heating. Three heating rods are inserted into circular holes in the copper plates that allow a maximum heating power of ${900}\,\text{W}$. The temperature of the sapphire glass surface is monitored by a type-K thermocouple at the surface recording five temperature readings per second and an infrared camera recording at 60 frames per second. The latter allows to control the uniformity of the surface temperature. The surface samples were sapphire glasses with a thickness of ${1}\,\text{mm}$ and a diameter of ${348}\,\text{mm}$. These surface samples could be plasma polymerized with a nanometric PTFE-like layer^22^ to obtain a static apparent contact angle of $\approx 120^{\circ }$. A temperature-resistant silicone oil (refractive index of 1.51) was used as a contact fluid between the surface sample and the prism to avoid total internal reflection in the air gap between the surface sample and the prism.

To qualitatively confirm the lower film thickness in the recess region and the movement of the fluid, tracer particles were added to the impacting droplet and the magnification of the bottom view was increased. To ensure that the tracers follow the flow reliably, a low Stokes number was required. Additionally, the particles should be significantly smaller than the residual film height. As the latter can be in the order of ${100}\,\upmu \text{m}$ without the hole and an even smaller height is assumed in the hole region, polystyrene particles (microParticles GmbH) with an average diameter of ${4.98}\,\upmu \text{m}$ were used.

### Image processing

MATLAB was used to analyze the impact processes. To correct for image distortions, images of a checkerboard calibration pattern were taken from each perspective. Using an automated detection algorithm, the vertical distortion of the bottom view, the rotation of the lateral and top view and the projective distortion of the top view were determined. The images displayed in this work are corrected for the determined distortions. The droplet diameters and impact velocities were automatically determined from the lateral view using binarized images after background subtraction. To determine the lamella height at the center, the double bubble shadow based Laser Pattern Shift Method^23^ was used. However, instead of using image binarization, a peak detection algorithm was used which was able to track the double bubble shadow, even when first signs of the curved lamella top appeared.

### Numerical simulations

The DNS are conducted using the multiphase flow solver Free Surface 3D (FS3D), which has already been applied successfully to various multiphase flow scenarios like drop film interactions^43^, drop impacts onto structured surfaces^39^, non-Newtonian jet breakup^44^, drop evaporation^45^ and thermocapillary flows^46^. FS3D solves the incompressible Navier-Stokes equations and the energy equation in temperature formulation on a MAC staggered grid and uses the Volume-of-Fluid (VOF) method to capture the interface^47^. For the interface reconstruction the Piecewise Linear Interface Calculation (PLIC) method is used^48^. The liquid volume, the momentum and the energy are advected conservatively with a second-order accurate Strang-Splitting approach^49,50^. Heat conduction as well as the viscous stress term in the momentum equation are solved implicitely. In the latter, the dynamic viscosity is treated as a temperature-dependent scalar by means of a four-parameter exponential correlation. The variable surface tension force is modeled as volume force by the balanced Continuous Surface Force (CSFb) method^51^. The Marangoni term in the momentum equation is calculated by using a gradient projection method. The corresponding gradient of the surface tension coefficient is obtained by means of the method that is already used for the calculation of the normal vector during the interface reconstruction. For the simulations, we use a three-dimensional computational domain extending to ${64}\,\upmu \text{m}$ in the vertical direction and ${512}\,\upmu \text{m}$ in the horizontal directions. The domain is discretized by an equidistant Cartesian grid with a resolution of $\Delta x={1}\,\upmu \text{m}$. On the bottom of the domain, a no-slip wall with a constant surface temperature is applied. Besides the two symmetry (slip-wall) boundary conditions which are used through the center of the bubble, homogeneous Neumann boundary conditions are applied on all remaining sides of the computational domain. The resolution of the computational grid has been investigated in a grid sensitivity study and the used resolution of 64 grid cells per bubble diameter has been considered to be sufficient. Furthermore, the used wide dimensions of the computational domain guarantee that the applied conditions on the domain boundaries do not influence the occurring deformation of the surface due to the thermocapillary convection.

### Supplementary Information

Supplementary Information 1.

Supplementary Information 2.

Supplementary Information 3.

Supplementary Information 4.

Supplementary Information 5.

Supplementary Information 6.

## Data Availability

Data supporting the findings of this study are available from P.P. upon request.
